# Comparison of the power of haplotype-based versus single- and multilocus association methods for gene × environment (gene × sex) interactions and application to gene × smoking and gene × sex interactions in rheumatoid arthritis

**DOI:** 10.1186/1753-6561-1-s1-s73

**Published:** 2007-12-18

**Authors:** Astrid Dempfle, Rebecca Hein, Lars Beckmann, André Scherag, Thuy Trang Nguyen, Helmut Schäfer, Jenny Chang-Claude

**Affiliations:** 1 Institute of Medical Biometry and Epidemiology, Philipps-University Marburg, 35037 Marburg, Germany; 2 Division of Cancer Epidemiology, German Cancer Research Center DKFZ, 69120 Heidelberg, Germany; 3 Institute of Medical Informatics, Biometry and Epidemiology, University of Duisburg-Essen, 45122 Essen, Germany

## Abstract

Accounting for interactions with environmental factors in association studies may improve the power to detect genetic effects and may help identifying important environmental effect modifiers. The power of unphased genotype-versus haplotype-based methods in regions with high linkage disequilibrium (LD), as measured by D', for analyzing gene × environment (gene × sex) interactions was compared using the Genetic Analysis Workshop 15 (GAW15) simulated data on rheumatoid arthritis with prior knowledge of the answers. Stepwise and regular conditional logistic regression (CLR) was performed using a matched case-control sample for a *HLA *region interacting with sex. Haplotype-based analyses were performed using a haplotype-sharing-based Mantel statistic and a test for haplotype-trait association in a general linear model framework. A step-down minP algorithm was applied to derive adjusted *p*-values and to allow for power comparisons. These methods were also applied to the GAW15 real data set for *PTPN22*.

For markers in strong LD, stepwise CLR performed poorly because of the correlation/collinearity between the predictors in the model. The power was high for detecting genetic main effects using simple CLR models and haplotype-based methods and for detecting joint effects using CLR and Mantel statistics. Only the haplotype-trait association test had high power to detect the gene × sex interaction.

In the *PTPN22 *region with markers characterized by strong LD, all methods indicated a significant genotype × sex interaction in a sample of about 1000 subjects. The previously reported R620W single-nucleotide polymorphism was identified using logistic regression, but the haplotype-based methods did not provide any precise location information.

## Background

The inclusion of gene × environment interaction terms in association analyses may improve the power to detect genetic effects and may contribute to the identification of important environmental effect modifiers [1]. Current technology makes it possible to genotype very dense sets of markers and the underlying genomic structure can be captured using haplotypes. The merits and drawbacks of haplotype-based compared with unphased genotype-based methods in the analysis of joint effects of genetic and non-genetic factors in regions with high linkage disequilibrium (LD, as measured by D') are of particular interest. When investigating candidate genes using dense markers that are in strong LD, the goal is to distinguish potentially causal variant(s) from those showing association merely due to LD. Using the Genetic Analysis Workshop 15 (GAW15) simulated data, we compared the power of three genotype- and haplotype-based methods to account for gene × environment interactions both to identify the genetic region and to identify the causal variant(s) in case-control scenarios. The methods are ordinary conditional logistic regression (CLR) and stepwise conditional logistic regression (SCLR) [2], Mantel statistics using haplotype sharing [3], and a haplotype-trait association statistic using a maximum likelihood estimate to test for interaction effects [4]. In contrast to the haplotype-trait association statistic, which provides a statistical test for each haplotype, CLR, SCLR, and the Mantel statistics provide a statistical test for each genetic marker. The methods also differ in the number of tests performed as well as in the number of degrees of freedom. To account for multiple hypotheses testing when investigating multiple single-nucleotide polymorphisms (SNPs) in a candidate gene and to obtain comparable power estimates, we applied a step-down minP algorithm [5].

In addition, we explored the performance of these methods in the GAW15 real data set for *PTPN22 *using markers in strong LD (in terms of D'). The data are a subset of the published data on rheumatoid arthritis (RA) [6]. RA is a complex autoimmune disease more common in women and in smokers [7] and with a moderately strong genetic component. Genetic association with RA was found in the *PTPN22 *gene [6,8,9], where the SNP R620W has been found to have a stronger effect in males than in females [6,10].

## Methods

### Simulated data sets

From the GAW15 simulated data modeling RA, we selected 100 replicate samples to represent a matched case-control study. For each replicate, the first affected offspring within an affected sibling pair of the first 500 families (1500 families in total) was chosen as case. Out of 2000 unrelated unaffected controls, 500 were matched to the cases by age and sex. The proportion of females:males in the case-control pairs was 2:1. In the simulated *HLA *region from the high-density scan on chromosome 6 with 18,000 SNPs, the disease loci DR and C have the same physical position and are in high LD. The DR locus has been simulated to have a strong effect on the risk of RA independently of sex, while locus C was generated to interact with sex, such that it increases risk only in women. We investigated these loci and the 10 flanking SNPs on either side, spanning a region of about 300 kb. The alleles of the multiallelic DR locus were recoded as biallelic based on the answers, i.e., the risk allele vs. all other alleles combined.

### Patients and genotypes in the RA sample

For the analysis of the real data set on *PTPN22*, we used all 1001 individuals from the NARAC (North American Rheumatoid Arthritis Consortium) sample for whom genotypes were available, these were 665 unrelated RA cases and 336 unrelated unaffected controls (Table 1, see Plenge et al. [6] for details of ascertainment). For the analysis of interaction effects of SNPs and smoking, we excluded individuals whose smoking status was not known. The control sample is not representative of the population regarding the distribution of sex and smoking because it includes a higher percentage of females and smokers than the case sample. Thus the estimated main effects of sex and smoking are strongly biased and not presented here. All 14 SNPs provided in *PTPN22*, which span approximately 58 kb, were investigated (Table 2).

**Table 1:** Descriptive statistics of the *PTPN22 *sample investigated

	Sex				Current smoking status						
			
	Male		Female		Non-smoker		Smoker		Unknown		Total
						
Sample	*N*	%	*N*	%	*N*	%	*N*	%	*N*	%	*N*
Control	41	12.2	295	87.8	118	35.1	52	15.5	166	49.4	336
Case	132	19.9	533	80.2	554	83.3	96	14.4	15	2.3	665

**Table 2:** *PTPN22 *analysis with LR and the Mantel statistic (adjusted *p*-values)

							Model 4^f^		Model 5^g^		Mantel^h^		
									
Marker	MAF^a^	D'^b^	*r*^2b^	Model 1^c^	Model 2^d^	Model 3^e^	Main	Int.	Main	Int.	I	II	III
rs3789604	0.20	1.00	0.04	0.97	0.96	0.96	0.95	0.95	0.97	0.94	0.40	**0.03**^i^	**<0.001**
rs3811021	0.20	0.95	0.04	0.97	0.96	0.97	0.95	0.95	0.97	0.95	0.40	**0.03**	**<0.001**
rs1217413	0.21	0.99	0.46	**0.05**	**0.026**	0.15	0.94	0.62	0.37	0.86	0.35	**0.02**	**<0.001**
ss38346942	0.01	0.60	0.00	0.95	0.96	0.97	1.00	1.00	0.97	0.97	0.35	**0.02**	**<0.001**
rs1217388	0.25	0.99	0.40	0.21	0.10	0.41	0.68	0.39	0.60	0.90	0.35	**0.02**	**<0.001**
ss38346943	0.03	0.05	0.00	0.81	0.88	0.97	0.95	0.96	0.28	0.49	0.40	**0.02**	**<0.001**
rs1310182	0.45	0.99	0.17	0.40	0.21	0.78	0.43	0.20	0.49	0.81	0.40	**0.03**	**<0.001**
ss38346944	0.02	0.19	0.00	0.97	0.96	0.91	0.94	0.94	1.00	1.00	0.40	**0.03**	**<0.001**
*R620W*	0.10	1.00	1.00	**0.004**	**0.001**	**0.02**	0.18	**0.01**	0.28	0.86	0.40	**0.02**	**<0.001**
rs12730735	0.30	0.94	0.05	0.81	0.74	0.78	0.95	0.94	0.97	0.94	0.40	**0.03**	**<0.001**
rs11102685	0.08	0.99	0.02	0.97	0.96	0.94	1.00	1.00	0.86	0.78	0.32	**0.03**	**<0.001**
rs12760457	0.29	0.97	0.06	0.90	0.88	0.87	0.93	0.81	0.97	0.92	0.38	**0.03**	**<0.001**
rs2488458	0.25	0.99	0.40	0.15	0.08	0.31	0.85	0.56	0.65	0.94	0.23	**0.03**	**<0.001**
rs1217414	0.26	1.00	0.05	0.69	0.47	0.97	0.74	0.54	0.09	0.21	0.23	**0.03**	**<0.001**

^a^Minor allele frequency among controls

^b^LD values calculated using the control population. All LR models consider one SNP at a time and include main effects of sex and smoking.

^c^Model 1, main effect

^d^Model 2, interaction term with sex

^e^Model 3, interaction term with smoking

^f^Model 4, main and interaction with sex

^g^Model 5, main and interaction with smoking

^h^Mantel statistics: I, main effect (*M*^0^); II, joint effect (*M*^1^) with sex; III, joint effect (*M*^1^) with smoking

^i^*p*-Values ≤ 0.05 are given in bold.

### Statistical analysis methods

#### 1. Logistic regression

We investigated four different sets of CLR models: 1) the main effect of each marker individually in 22 separate regression models; 2) only the joint effect of each genotyped marker individually with sex as an interaction term. This approach was considered in order to compare CLR and the Mantel statistics for joint effects, *M*^1^, mentioned below; 3) both the main effect of each marker individually and their interaction with sex; 4) SCLR (stepwise CLR with backwards model selection based on the Akaike information criterion) with the full model including all main and first-order interaction effects of the 22 markers simultaneously. The SCLR approach was proposed specifically to distinguish between causal variants and those merely in LD [2]. Note that these analyses only use unphased multilocus genotype data. We used an additive genetic model on the logit scale, i.e., multiplicative on the odds ratio scale, without dominance effects to reduce the number of degrees of freedom.

We employed analogous unconditional LR models for the real data on *PTPN22 *because cases and controls were not matched. Here, gene × environment (current smoking) and gene × sex interactions were investigated. All CLR and LR analyses were performed using the computer program R (version 2.3.1), using the general linear model (glm) procedure and the step function for stepwise model selection.

#### 2. Haplotype-based analysis

##### 2.1 Mantel statistic using haplotype sharing

We applied the approach of Mantel's statistics for space-time clustering to correlate genetic and phenotypic similarity [3]: $M^{0}(x):=\sum_{i<j}L_{ij}(x)Y_{s_{i}s_{j}}$ whereby *x *denotes a putative disease locus, *i *and *j *are haplotypes, L_*ij*_(*x*) is measured as the number of intervals surrounding *x *flanked by markers identical by state (haplotype sharing). The sum is over all pairwise comparisons of haplotypes. $Y_{s_{i}s_{j}}$ denotes the phenotypic similarity of the individuals *s*_*i *_and *s*_*j*_, and is defined as the mean-corrected product $Y_{s_{i}s_{j}}=(y_{s_{i}}-\mu_{y})(y_{s_{j}}-\mu_{y})$, where *μ*_*y *_denotes the sample mean, and $y_{s_{i}}$ and $y_{s_{j}}$ the disease status of *s*_*i *_and *s *_*j*_. To analyze the joint effect of a marker locus and an environmental factor, a measure of exposure similarity $Z_{s_{i}s_{j}}$ was introduced: $M^{1}(x):=\sum_{i<j}L_{ij}(x)Y_{s_{i}s_{j}}Z_{s_{i}s_{j}}$. $Z_{s_{i}s_{j}}$ is computed as $Z_{s_{i}s_{j}}$ = 1, ${\mathrm{if z}}_{s_{i}}$ = $z_{s_{j}}$, and $Z_{s_{i}s_{j}}$ = 0 else, where $z_{s_{i}}$ and $z_{s_{j}}$ denote the environmental factor of *s*_*i *_and *s*_*j*_. *M*^1 ^is a test for joint effects and does not distinguish between genetic main effects and interaction effects.

##### 2.2 Haplotype-trait association

We applied a test for haplotype-trait association in a general linear model framework, using maximum likelihood estimates for main and interaction effects of haplotypes and non-genetic factors, allowing for haplotype phase to be ambiguous [4,11]. Two regression models were applied. The first model contained sex and all main effects of haplotypes. The second model included all main effects and first-order haplotype-sex interaction effects. The effects of the haplotypes were modeled as additive. We first used the most frequent haplotype, which included the risk alleles on the loci DR and C, as baseline category. We also analyzed the data using a less frequent haplotype, which did not include any risk alleles, as the baseline haplotype. Certainly, without prior knowledge of the answers, we would not have done so. Only haplotypes with a frequency of at least 5% were considered. We modified the function haplo.glm, which is included in the haplo.stats R-library [4].

For the *PTPN22 *data set, we used a model which included the main effects of the haplotypes, sex, and smoking, as well as the first-order haplotype-sex or haplotype-smoking interaction terms.

#### 3. Permutation procedure and step-down minP adjusted *p*-values

The numbers of tests and degrees of freedom differed between the statistical methods and models. Thus, we permuted the case-control status while keeping together genotypes and sex-as well as smoking status in the analysis of the real data-for each individual, and calculated adjusted *p*-values by a step-down minP algorithm [5].

## Results

### Simulated data

As depicted in Table 3, power of CLR was generally high for Model 1 to detect the genetic main effect at the DR locus and for Model 2 to detect the joint effect of the SNPs and sex. For SNPs 5 and 15 only, there was hardly any power. In contrast, modeling of both a genetic main effect and an interaction effect resulted in very low power to detect the interaction and low to moderate power to detect the genetic main effect. SCLR performed highly unsatisfactorily and had very low power for all effects modeled.

**Table 3:** Power for the CLR and Mantel statistic using 500 case-control pairs

						Model 3^e^		Model 4^f^		Mantel	
								
	MAF^a^	D'^b^	*r*^2b^	Model 1^c^	Model 2^d^	Main	Interaction	Main	Interaction	M^(0) ^(main effect)	M^(1) ^(joint effect)
SNP1	0.32	1	0.16	1	1	0.28	0	0.02	0.01	1.00	1.00
SNP2	0.12	1	0.04	0.99	0.99	0.06	0	0.01	0.01	1.00	1.00
SNP3	0.37	0.95	0.17	1	1	0.48	0.01	0.02	0.01	1.00	1.00
SNP4	0.49	1	0.34	1	1	0.37	0	0.02	0.01	1.00	1.00
SNP5	0.05	0.21	0	0.01	0.01	0	0.01	0.01	0.01	1.00	1.00
SNP6	0.13	1	0.05	1	1	0.08	0	0.01	0.01	1.00	1.00
SNP7	0.13	1	0.05	1	1	0.08	0	0.01	0	1.00	1.00
SNP8	0.23	1	0.1	1	1	0.36	0	0.02	0.02	1.00	1.00
SNP9	0.23	1	0.1	1	1	0.36	0	0	0	1.00	1.00
SNP10	0.41	0.94	0.41	1	1	0.51	0.01	0.05	0.01	1.00	1.00
DR locus	0.25	1	1	1	1	0.44	0.01	0.14	0.01	1.00	1.00
Locus C	0.42	1	0.45	1	1	0.2	0.01	0.04	0.01	1.00	1.00
SNP13	0.4	1	0.22	1	1	0.42	0.01	0.09	0.02	1.00	1.00
SNP14	0.33	0.99	0.65	1	1	0.4	0.03	0.03	0.01	1.00	1.00
SNP15	0.48	0.93	0.3	0.15	0.07	0	0	0.01	0.01	1.00	1.00
SNP16	0	0.92	0	1	1	0.56	0	0.02	0.01	1.00	1.00
SNP17	0.24	0.98	0.92	1	0.99	0.03	0	0.01	0.01	1.00	1.00
SNP18	0.1	1	0.04	1	0.99	0.07	0	0.01	0.01	1.00	1.00
SNP19	0.12	1	0.05	1	1	0.12	0	0.01	0.01	1.00	1.00
SNP20	0.39	0.56	0.07	1	1	0.52	0	0.01	0.01	1.00	1.00
SNP21	0.47	0.94	0.26	1	1	0	0.02	0.01	0.01	1.00	1.00
SNP22	0.1	0.71	0.02	1	1	0.33	0	0.07	0.01	1.00	1.00

Power is the percentage of replicates in which the main or interaction effect of the respective marker had an adjusted *p*-value ≤ 0.05.

^a^Minor allele frequency among controls

^b^LD values calculated using the control population in Replicate 1

^c^Model 1, main effect of one SNP at a time

^d^Model 2, interaction term with sex of one SNP at a time

^e^Model 3, main and interaction with sex of one SNP at a time

^f^Model 4, stepwise model with main and interaction effects of all SNPs

The haplotype-sharing-based Mantel statistics had 100% power for all markers both for the genetic main effect and for the joint effect (Table 4) even when no more than 50 case-control pairs were investigated (data not shown). For the haplotype-trait association test, we present results only for the four haplotypes, which were observed in at least 80 of 100 samples (Table 2). The GAW15 data were simulated such that the allele coded 3 at the DR locus increases RA risk while the allele coded as 1 at locus C was simulated to increase risk for RA only in women. Thus it was surprising that both risk-related alleles were also included in the most common haplotype, the reference haplotype by default. All three haplotypes indicated the main and the interaction effect with power estimates ranging between 0.82 and 1, and 0.65 and 0.78, respectively. We reexamined the data using as reference the second most frequent haplotype, which did not comprise the risk alleles. The estimated power was moderate to high for the detection of the main effect (0.57 to 1) and low for the detection of the interaction effect with sex (0.08 to 0.51, data not shown).

**Table 4:** Power of the haplotype-trait association test using 500 case-control pairs

Common haplotypes^a^	Frequency^b^	Model 1^c ^(main effects of haplotypes)	Model 2^c ^(main effects and haplotype-sex interaction effects)	
			
			Main	Interaction
2122111112*1*22221211221	0.06	1	0.95	0.78
**1111111111*3*11111111211**	**0.07**	**0.99**	**0.82**	**0.66**
2122111112*1*22221211121	0.11	1	0.99	0.65
**1111111111*3*11111111111 (reference haplotype)**	**0.39**			

^a ^Haplotypes present in at least 80 replicates of the 100 replicates (risk loci are underlined, DR locus italicized, C locus not italicized), including the alleles at the 22 loci

^b ^Estimated haplotype frequencies (combined sample) in the 100 replicates

^c ^Power is the percentage of replicates in which the main or interaction effect of the respective haplotype had an adjusted *p*-value of ≤ 0.05.

### Real data

In the *PTPN22 *region, LR identified significant effects at R620W (*p *= 0.04) and rs1217413 (0.05), considering main effects only and when considering also interaction effects with sex (*p *= 0.007 and *p *= 0.026, respectively) (Table 2). The remaining SNPs surrounding R620W did not show significant results. An interaction effect with smoking was not observed. As observed for the simulated data, power was low for all effects modeled using stepwise LR (data not shown).

The Mantel statistics did not yield significant main effects of the investigated SNPs with the lowest adjusted *p*-value at 0.23 (Table 2). However, evidence for joint effects (*M*^1^) both with sex and with smoking was found (lowest adjusted *p*-values of 0.02 and <0.001, respectively). Six common haplotypes with frequencies ≥ 5% were estimated in *PTPN22 *(Table 5). Applying the haplotype-trait association test, one haplotype detected with a frequency 13.6% was associated with a significantly increased risk in the model comprising only main effects. This haplotype differed from all other haplotypes at locus R620W (Table 5). One haplotype with frequency 8.52%, which was found to be significant in the haplotype-sex analysis, was associated with a decreased risk. Compared to the reference haplotype, this haplotype carries the same allele at R620W, but differs for SNPs rs12730735 and rs1217414. In both cases, the respective alleles are also present on other haplotypes, which do not show an interaction, thus a specific interacting disease variant could not be identified. There was no evidence for a haplotype-smoking interaction.

**Table 5:** Adjusted *p*-values for the haplotype-trait association test in *PTPN22*

			Model 2 (main effects and haplotype-sex interaction effects)		Model 2 (main effects and haplotype-smoking interaction effects)	
				
Common haplotypes in the investigated regions^a^	Frequency	Model 1 (main effects of haplotypes)	Main	Interaction w/sex	Main	Interaction w/smoking
3 3 1 1 1 1 1 2 **3 **4 4 2 2 3	0.19	0.25	0.67	0.58	0.97	1
4 1 1 1 1 1 3 2 **3 **4 2 2 2 1	0.09	0.2	0.89	0.98	0.91	0.78
4 1 1 1 1 1 3 2 **3 **4 4 2 2 1	0.09	0.44	0.07	**0.04**^b^	0.91	0.93
4 1 3 1 3 1 1 2 **1 **4 4 2 4 3	0.12	**<0.001**	0.6	0.15	0.17	0.82
4 1 3 1 3 1 1 2 **3 **4 4 2 4 3	0.12	0.44	0.98	0.98	0.97	1
4 1 1 1 1 1 3 2 **3 **2 4 4 2 3	0.27					
**(reference haplotype)**						

Loci in the table are listed as described in Table 4.

^a^Established risk locus R620W is bold, underlined

^b^*p*-Values ≤ 0.05 are given in bold.

## Discussion

Current technology permits genotyping of dense marker sets across the whole genome. Once associated regions have been identified, efforts will be made to confirm these results in independent samples by a hypothesis-based candidate gene approach in which control of the type I error rate will be of great importance. Accounting for gene × environment interaction given moderate genetic main effects may improve the power to detect or confirm an association with a gene, a genetic region, or causal variant(s). Given this scenario, we investigated the performance of some methods proposed for detecting genetic main and gene × environmental interaction effects. We found that stepwise LR or CLR performed poorly given strong LD (as measured by D') between markers-a phenomenon called (multi-)collinearity that results in overfiting of the model and weak robustness [12]. It is well known that automated stepwise methods do not necessarily produce the best model if there are redundant predictors; often the model created will have poor predictive accuracy [13-15]. Although stepwise model selection was proposed for elucidating which of several markers in LD might be functionally relevant [2], this strategy seems to be inefficient when strong LD (in terms of D') exists. In this situation, ordinary LR performs very well when only the main effect of each marker is tested separately in a model and the *p*-values are adjusted for the total number of tests performed. The low power for SNP 5 is likely due to low LD (*r*^2 ^≈ 0) with the disease loci and non-matching allele frequencies [16]. This does not appear to apply for SNP 15, where power is low but LD is stronger. All other markers had a power of about 100% for the genetic main effect despite mostly low *r*^2^-values between these markers and the disease loci. Therefore, in the simulated scenario, it was not possible to localize the functional variant within this region, e.g., there was no general tendency for the true causal locus to have the smallest *p*-value or largest effect size estimate. The simulated genetic main effect was also much stronger than the interaction effect, so that there was no improvement adding a gene × sex interaction term in the model. A much larger sample size would have been necessary for detecting the interaction effect.

There was only a limited number of haplotypes estimated from the 22 simulated markers due to the strong LD (as measured by D') between them. Both haplotype-based methods were able to detect association in the investigated region but they failed to identify the disease variant(s). The findings regarding joint effects suggest that analysis for joint effects may be an option for detecting genetic effects in some situations. For smaller sample sizes, the Mantel statistics for joint effects (*M*^1^), however, is more powerful than a similar LR modeling of interaction effects (data not shown).

For the analysis of *PTPN22 *in RA, we used a subset of the data of Plenge et al. [6], who identified an interaction of R620W with sex in a case-only analysis in their complete data set of about 4100 RA cases and controls. Using the different LR models, we were also able to detect this genotype × sex interaction in a subsample of 1001 subjects at R620W. The lack of significance for the genetic main effect in the model including both main and interaction terms, indicated that the effect of R620W indeed depends on the gender of the genotype carrier. Furthermore, it was possible to distinguish R620W from neighboring markers with allele frequencies that did not match and low *r*^2^-values [16]. The Mantel statistic failed to identify main effects but showed strong evidence for a joint effect both with gender and with smoking in model *M*^1^. These significant effects may however be due to apparent strong main effects of sex and smoking caused by an unrepresentative control sample and may not reflect a true interaction effect. No specific marker could be detected as disease-causing polymorphism because of the dependency of the test statistics at different markers. By contrast, the results of the haplotype association method haplo.glm were similar to the results of the LR models implicating the R620W locus as a risk locus for RA. However, it is unclear whether the significant haplotype-sex interaction observed for a different haplotype refers to a further disease variant.

## Conclusion

Our results suggest that stepwise or other automated variable selection methods are not suitable for the investigation of gene × environment interactions in regions with high LD, as measured by D'. Given strong genetic main effects and moderate gene-sex interaction, as in the simulated data, both simple CLR and haplotype-based methods have adequate power for the detection of genetic main effects without considering gene × sex interaction in moderate sample sizes. In the simulated scenario, haplotype-trait association tests had better power than simple LR modeling to detect moderate gene × sex interactions. However, this result cannot be generalized to other situations without further simulation studies under different genetic models. On the other hand, haplotype-based methods that always use data from consecutive markers tend to yield similar results for neighboring markers, thus making localization of a causal variant potentially more difficult than with genotype-based methods that consider each marker at a time. Thus, the potential for localization of causal variants with each method should also be explored in further simulation studies.

## Competing interests

The author(s) declare that they have no competing interests.
